# Superhydrophobic 304 Stainless Steel Mesh for the Removal of High-Density Polyethylene Microplastics

**DOI:** 10.1021/acs.langmuir.2c00803

**Published:** 2022-04-25

**Authors:** Oriol Rius-Ayra, Alisiya Biserova-Tahchieva, Victor Sansa-López, Núria Llorca-Isern

**Affiliations:** CPCM Departament de Ciència dels Materials i Química Física, Facultat de Química, Universitat de Barcelona, Martí i Franquès 1-11, 08028 Barcelona, Spain

## Abstract

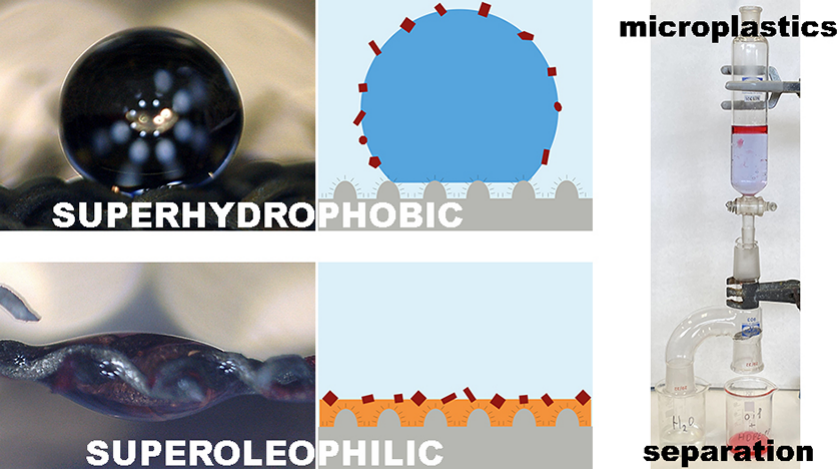

Microplastics are a global issue that affects the environment, economy, as well as human health. Herein, we present a superhydrophobic 304 stainless steel mesh obtained by chemical etching followed by a liquid-phase deposition of lauric acid that can be used for microplastic removal. Field emission scanning electron microscopy (FE-SEM) and high-resolution X-ray photoelectron spectroscopy (HR-XPS), among other techniques, were used to identify the hierarchical structure and chemical composition of the surface. They revealed that iron laurate decreased the surface free energy. The 304 stainless steel mesh was superhydrophobic (169°) and superoleophilic (0°). Taking advantage of these wetting properties, we showed an innovative use of these superhydrophobic surfaces in the removal of microplastics. Additionally, we analyzed the removal efficiency from a surface and colloidal point of view that allowed us to explain and clarify why microplastics can also be removed by their wetting properties. The loss of a double electrostatic cloud between the microplastics and the predominance of van der Waals interactions in the organic phase promote the removal of these persistent pollutants from water.

## Introduction

Microplastics (MP) are defined by their size (<5 mm) and are an emerging type of debris that can be found in rivers, oceans, the atmosphere, soil, and even in animals and humans.^1−3^ Additionally, these types of solid pollutants, which have different chemical compositions with polyethylene MP being the most abundant type (54.5%),^4,5^ can behave as a vector for metals and metalloids that are adsorbed on to the polymer surface.^6−8^ This issue has attracted a lot of interest in different scientific fields performing research that aims to prevent, reduce, and remove these pollutants from the environment.^9−12^ The methodologies used to remove MP can be classified into two main groups: the more traditional methods, such as coagulation and filtration,^13−15^ and emerging ones that remove MP with extremely high efficiencies.^16−18^ Some noteworthy methods are photocatalytic micromotors such as BiVO_4_/Fe_3_O_4_^19^ and Au@Ni@TiO_2_,^20^ surface-functionalized microbubbles,^21^ froth flotation,^22^ and surface functionalization of the MP with hypochlorite (ClO^–^)^23^ or magnetite (Fe_3_O_4_).^24^

From a surface point of view, MP are widely found in water. Thus, their wetting properties have to be considered not only to fully understand their behavior in water but also to be used carefully and taken into account during their removal. In fact, due to their chemical composition, which is primarily C–H bonds like in polyethylene ((−CH_2_–CH_2_−)_*n*_) or polypropylene ((−CH_2_–CH(CH_3_)−)_*n*_), MP display water contact angles (WCAs) greater than 90°, leading to hydrophobicity that increases the difficulty of removing them from water. In this scenario, superwettable materials, particularly superhydrophobic/superoleophilic surfaces, present a key role in the capture and removal of these solid pollutants. It is well-known that superhydrophobic materials are defined by a WCA > 150° as well as a sliding angle (SA) and contact angle hysteresis (CAH) of less than 10°.^25,26^ Additionally, superhydrophobic surfaces can be classified into different wetting states according to the adhesive force, such as the rose petal effect (high adhesive force) or lotus leaf effect (low adhesive force).^27,28^ Three models have been used to explain the surface wettability:^29^ Young’s model that connects the WCA with the surface free energy;^30−32^ Wenzel’s model that considers the contribution of the surface roughness leading to an homogeneous regime;^33^ and the Cassie–Baxter model that explains the heterogeneous regime where an air interface is found between the solid and the liquid.^34^ Moreover, these surfaces can be used to separate oil from water mixtures as well as oil-in-water emulsions with extremely low oil contact angles (OCAs) close to 0°.^35−37^ Recently, the ability of superhydrophobic materials to remove microplastics is attracting attention. A few articles show these innovative applications,^38−40^ but the removal mechanism has to be exhaustively discussed considering the wetting properties from a chemical and physical point of view.

Herein, we used a 304 stainless steel (SS) mesh that has not been extensively used before because the stainless steel was not identified^41−43^ or different types of SS were slightly studied such as 316L SS^44^ or 304 SS.^45^ Superhydrophobicity was conferred to the mesh by chemical etching (FeCl_3_/HCl/H_2_O_2_) followed by a liquid-phase deposition of lauric acid. The 304 SS mesh showed a WCA of 169° and an OCA of 0°. Taking advantage of these wetting properties, we studied its ability to remove high-density polyethylene MP from water. The mesh had a removal efficiency of 99%, also showing high reusability. Finally, considering the relationship between the wetting properties of the MP and the superhydrophobic mesh, we describe the removal mechanism from a colloidal and surface point of view based on the binding energy of the solid pollutant and the Derjaguin–Landau–Verwey–Overbeek (DLVO) theory.

## Experimental Section

### Superhydrophobic Mesh

A 304 SS mesh with a pore size of 0.5 mm^2^ and a filament diameter of 270 μm was used as a substrate. It measured 2 × 3 cm^2^ in size. The substrates were cleaned with deionized water, sanded with SiC P180 abrasive paper, and rinsed again with water. Then, the substrates were etched for 10 min using a 2 M FeCl_3_ solution containing deionized water, 37% HCl (w/w), and 5% H_2_O_2_ (w/w) at a ratio of 15:1:1 (all reactants purchased from Scharlau). The meshes were then rinsed in water and dried in an oven at 55 °C for 1 h. After that, the etched substrates were immersed for 20 min in a solution containing 0.15 M extra pure lauric acid (99.8% from Scharlau) in 96% v/v absolute ethanol (synthesis grade from Scharlau) at 60 °C. Finally, the treated substrates were cleaned with absolute ethanol and dried in an oven at 55 °C for 45 min.

### Durability Tests

The durability of the superhydrophobic 304 SS mesh was evaluated by two methods. One of these methods involved placing aqueous solution droplets of different pH values in acid or alkali solution (pH = 0, 1, 4, 7, 9, 12, and 14 using H_2_SO_4_, HNO_3_, CH_3_COOH, H_2_O, NH_3_, and NaOH, respectively) on the mesh surface for 10 min. Additionally, hexane was used to confirm that the superhydrophobic mesh did not lose its wetting properties after being in contact with the organic solvent. The other method involved performing an abrasive grinding paper test to study durability under severe abrasive conditions. The superhydrophobic 304 SS mesh was placed against and moved 10 cm along the SiC P1200 grinding paper for 10 cycles under a constant load of 5 kPa. After each cycle, the surface was cleaned with forced air before the WCA and SA were measured.

### MP Removal

High-density polyethylene (HDPE) MP (purchased from Abifor) with sizes of 133 ± 34 and 200 ± 20 μm were used. These MP were removed from a 3.5% (w/w) NaCl aqueous solution (pH = 7.00). Then, 50 mL of the aqueous solution was prepared to further add 20 mg of the MP. After that, 10 mL of hexane (purchased from Scharlab) were added to the mixture and stirred. The aqueous phase was colored with thymol blue and the hexane was colored with Oil Red O biological stain (both from Scharlab) to clearly show both phases. The separation was carried out in a laboratory-made device that consisted of a dropping funnel (where the solution containing both phases (organic and aqueous) was poured through at a flux of 3 mL/s), an inverted Claisen adapter (where the 304 SS mesh was located at a tilt of 5° corresponding to the SA), and two beakers to collect the two separated phases. After that, the mesh was dried in a fume hood (55 °C) to weigh the mass of the HDPE-MP retained in the mesh as well as the mass of HDPE-MP collected in the beaker. This procedure was repeated three times, with the superhydrophobic substrate washed with ethanol after each test. The added mass of HDPE-MP before and after the removal process was controlled in order to determine the removal efficiency (η). This value was measured by weighing the MP mass before (10, 15, and 20 mg) and after the removal process by weighing the HDPE-MP collected in the beaker as well as those retained in the superhydrophobic mesh.

### Characterization Techniques

Different characterization techniques were used to determine the morphological and chemical composition, which are key parameters to determine the superhydrophobicity of samples. The surface was characterized on a JEOL J-7100 field emission scanning electron microscope (FESEM) to study its detailed morphology. Energy-dispersive X-ray spectroscopy (EDS) was used to determine the semiquantitative elemental composition of the samples that had been carbon sputtered. High-resolution X-ray photoelectron spectroscopy (HR-XPS) was performed on a PHI ESCA-5500 system using a monochromatic X-ray source (Kα(Al) = 1486.6 eV and 350 W) to determine the chemical composition of the system. Attenuated total reflectance Fourier transform infrared (ATR-FTIR) spectroscopy was performed on a Fourier Bomem ABB FTLA system in the range of 4000–525 cm^–1^ at a resolution of 4 cm^–1^ to determine the presence of hydrocarbon acid and its chemical bonds. The sustainability of the removal process was studied by determining the chemical composition of an aqueous solution (1% HNO3 to avoid the precipitation of metallic oxides) that had been in contact with the superhydrophobic mesh. For this purpose, inductively coupled plasma-optical emission spectroscopy (ICP-OES, PerkinElmer Optima 3200RL) was undertaken. Static WCA, CAH, and SA images were taken using the sessile method involving a Levenhuk digital microscope and 3.5 μL of deionized water at room temperature. Hexane (purchased from Panreac) was used to measure the OCA. In the case of HDPE-MP, contact angle measurements were performed as follows. MP were sprinkled over a glass slide containing an adhesive before being flattened by another glass slide to prevent roughness effects, with the excess powder removed.^46^ The ImageJ software was used to measure all contact angles. The reported values of contact angle measurements were the average of three measurements of droplets at different parts on the surface.

## Results and Discussion

### Surface Characterization

**Figure 1:**
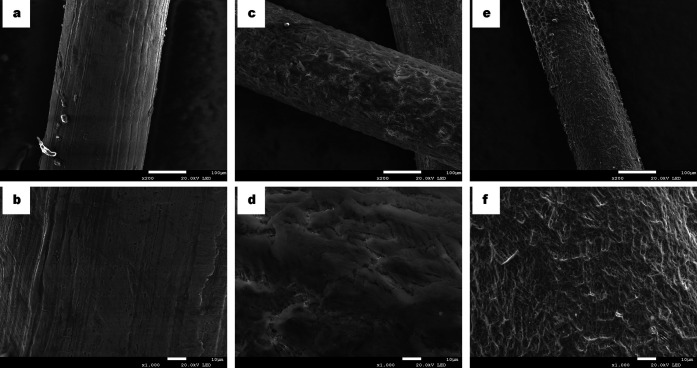
FESEM micrographs of the 304 SS mesh and its magnification: (a, b) untreated 304 SS mesh without the presence of a coating layer; formation of a coating layer (c, d) after chemical etching and (e, f) after lauric acid modification.

FESEM micrograph analysis was carried out to study the surface morphology of the superhydrophobic mesh (Figure 1). As seen in Figure 1a and b, the untreated 304 SS mesh did not present any type of coating and the roughness was caused by cleaning the mesh with abrasive paper. After the chemical etching with the FeCl_3_/HCl/H_2_O_2_ aqueous solution, a coating was formed throughout the whole surface of the 304 SS mesh, showing a heterogeneous rough surface formed by cavities (Figure 1c, d). Finally, after the liquid-phase deposition of lauric acid in ethanol, the surface remained coated and also presented cavities (Figure 1e, f). Additionally, semiquantitative EDS (wt %) showed the presence of 15.9% Fe (Kα = 6.4 eV), 21.2% O (Kα = 0.5 eV), 20.5% C (Kα = 0.277 eV), 7.6% Mn (Kα = 5.9 eV), 5.6% Cr (Kα = 5.4 eV), 1.8% Cu (Kα = 8.1 eV), and 1.4% Ni (Kα = 7.5 eV) in addition to other minor elements (P, Si, and S) (Figure S1). These results demonstrated the formation of a thin layer composed of iron, oxygen, and also carbon as the major elements.

While the FESEM micrographs showed that the surface of the 304 SS mesh had been chemically etched, EDS analysis revealed that the surface had been clearly modified. The mesh surface was coated by an oxide layer (Fe_*x*_O_*y*_). The chemical etching caused the formation of different structures at the microscale and nanoscale, leading to a hierarchical structure that is a key feature of the Cassie–Baxter heterogeneous wetting state.^34^

### Chemical Characterization

**Figure 2:**
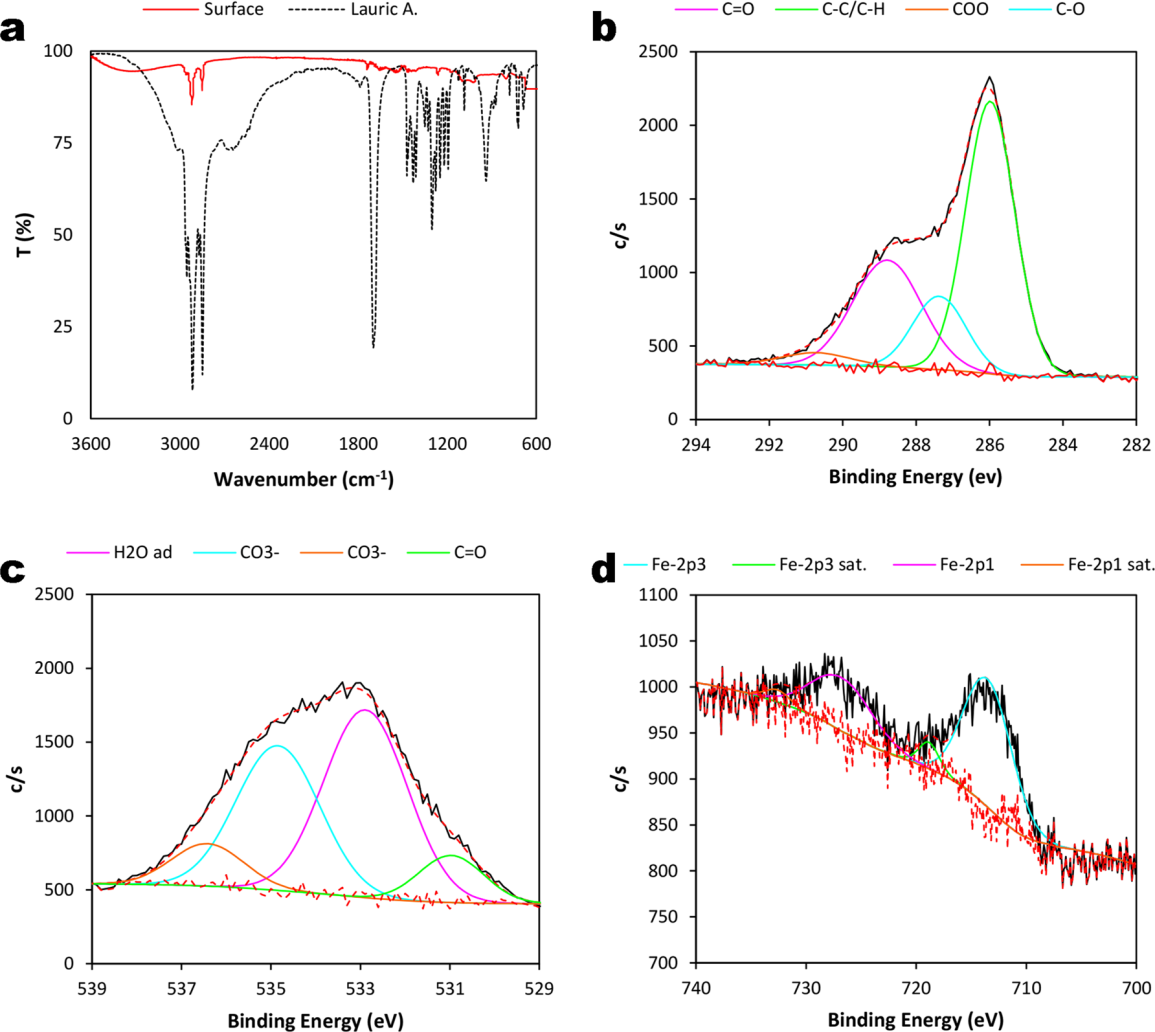
Surface chemical composition: (a) ATR-FTIR spectroscopy of the superhydrophobic mesh revealing the presence of a carboxylate group. HR-XPS analysis corresponding to (b) C-1s, (c) O-1s, and (d) Fe-2p.

The chemical composition of the samples at the surface level was also determined to explain the wetting properties of the modified mesh. ATR-FTIR spectroscopy was performed to establish the presence of chemical bonds at the surface level of the modified 304 SS mesh (Figure 2a). There were three consecutive signals on the respective spectra of the modified mesh and pure lauric acid between ca. 3000 cm^–1^ and ca. 2800 cm^–1^ that were attributed to v_as_CH_3_, v_as_CH_2_, and v_s_CH_2_–CH_2_, respectively, from the sp^3^ carbon atoms of the alkyl chain.^47−49^ The first significant strong band for the carbonyl group of lauric acid was on both spectra next to ca. 1700 cm^–1^ corresponding to vC=O. Two bands on the spectrum for pure lauric acid close to ca. 1300 cm^–1^ and ca. 1200 cm^–1^ corresponded to different vO–H bonds. The first one corresponded to the carboxylic group of lauric acid, while the second one corresponded to the oxide layer on the modified substrate. A signal next to ca. 1300 cm^–1^ on the spectrum of pure lauric acid assigned to vC–O was not present on that of the superhydrophobic substrates, confirming the formation of the carboxylate, with both C–O bonds becoming equal because of the electronic resonance between them.^50,51^ Accordingly, the band at ca. 1350 cm^–1^ on the spectrum of the prepared mesh was attributed to v_s_COO. The signal on the spectra for both the prepared substrate and the pure acid at ca. 1450 cm^–1^ was assigned to δCH_2_. HR-XPS was also used to determine the chemical states of C-1s, O-1s, and Fe-2p at the surface level. For C-1s (Figure 2b), there were four different deconvolutions at 286, 287, 288, and 290 eV assigned to the C–C/C–H bond, and C–O, C=O, and O–C=O as the carboxylate functional groups, respectively.^52^ In the case of Fe-2p (Figure 2c), there were four deconvolutions at 713 and 726 eV assigned to Fe-2p3/2 and Fe-2p1/2 that corresponded to different phases of iron oxide: γ-Fe_2_O_3_ for the signal at 713 eV and α-FeOOH for the signal at 726 eV. Additionally, more peaks of lower intensity were detected corresponding to the satellites of Fe-2p3/2 (719 eV) and Fe-2p1/2 (732 eV).^53−55^ Finally, for O-1s (Figure 2d), there were four deconvolutions at 531, 532, 534, and 536 eV. The first and second deconvolutions were assigned to the C=O bond and adsorbed water at the surface level, respectively, while the third and fourth deconvolutions were attributed to the presence of a carbonate-like chemical species.^53,54,56^

Surface chemical modification can be described as a combination of a comproportionation process and a subsequent liquid-phase deposition of lauric acid onto the oxidized layers. First, the comproportionation involves a redox reaction that causes the oxidation of the 304 SS mesh surface and the reduction of H_2_O_2_ in the HCl media. In fact, it is the Fe(III) ions that target the microscopic defects of the 304 SS mesh caused by the abrasive paper, penetrating into the surface to produce the observed roughness. The redox reaction can be described as follows:
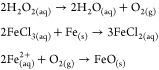
During this reaction, part of the surface iron is oxidized to generate FeCl_2_ in the aqueous solution, which also forms FeO on the 304 SS mesh. As iron(III) oxides are the most thermodynamically stable oxides, the surface is oxidized in the presence of atmospheric oxygen and humidity as follows:
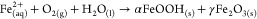
Once the surface was etched and the maghemite (γ-Fe_2_O_3_) and goethite (α-FeOOH) layers were formed, the mesh was washed several times to remove the reactants before the liquid-phase deposition of lauric acid in an ethanol solution, as follows:
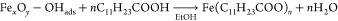
The carboxylic functional group of lauric acid (−COOH) reacts with the OH^–^ adsorbed on to the Fe_*x*_O_*y*_ surface (Fe_*x*_O_*y*_ represents the iron oxide or oxide-hydroxide) that presents certain alkaline characteristics, leading to the formation of the iron laurate as a chelate and the release of water molecules.

### Wetting Properties

**Figure 3:**
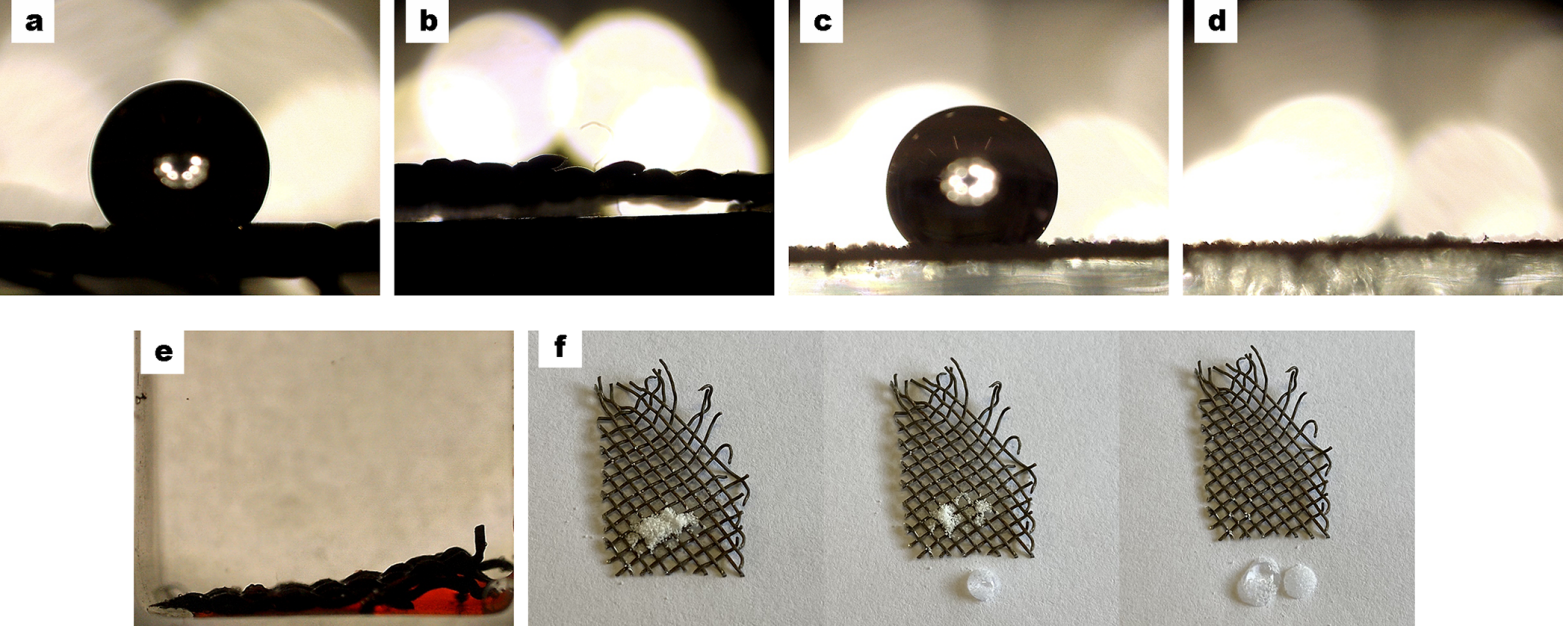
Contact angle measurements: 304 SS mesh after the liquid-phase deposition of lauric acid showed (a) a WCA of 169 ± 1° and (b) an OCA of 0°. In the case of the HDPE-MP powder, (c) the WCA was 136 ± 2°, (d) the OCA was 0°, and (e) the UWOCA (3 ± 1°), showing underwater superoleophilic properties with total oil sorption. (f) Self-cleaning properties of the superhydrophobic 304 SS mesh after the addition of HDPE-MP powder.

It is well-known that contact angle measurements are of significant importance in defining the wetting properties of a surface (Figure 3). Herein, we determined the WCA, SA, CAH, and OCA of the etched 304 SS mesh as well as of the substrate modified with lauric acid. Additionally, the WCA and OCA for the HDPE-MP were measured. The WCA for the etched 304 SS mesh was 37 ± 2°, indicating hydrophilic properties. After the liquid-phase deposition of lauric acid, the WCA was 169 ± 1°, revealing superhydrophobic properties (Figure 3a) with an SA of 5 ± 1° and a CAH of 4 ± 1°. The bare 304 SS mesh (untreated mesh) had a very similar WCA as that of the etched substrates. In the case of the superhydrophobic mesh, the OCA was 0°, showing superoleophilic properties (Figure 3b). The wetting state of the superhydrophobic 304 SS mesh was assigned to the well-known Cassie–Baxter model.^34^ In the case of HDPE-MP, the WCA was 136 ± 2° (Figure 3c) and the corresponding OCA was close to zero, showing superoleophilic properties (Figure 3d). The underwater oil contact angle (UWOCA) was 3 ± 1°, with total oil sorption (Figure 3e). Moreover, self-cleaning properties were demonstrated by adding HDPE-MP powder onto the superhydrophobic surface and removing it with just a few water droplets at a 5° tilt (Figure 3f).

As described before, the combination of chemical etching and liquid-phase deposition changed the wetting properties of the 304 SS mesh from hydrophilic to superhydrophobic. These properties changed due to the presence of the hierarchical structure that, combined with iron laurate, caused a decrease in the surface free energy of the system due to the long chain of the fatty acid, which caused the cavities to be filled with air instead of water, thereby conferring superhydrophobicity. At the same time, the surface was also superoleophilic with hexane as the organic phase. This is because the long carbon chain of iron laurate has more affinity for oil. Therefore, hexane can be trapped in the cavities, even under water where air cavities can be filled with oil. In the case of HDPE-MP, the wetting properties were directly related to their intrinsic nature, that is, the presence of the C–H chain that confers hydrophobicity, but presents superoleophilicity due to the higher affinity for hexane.

The durability of the superhydrophobic 304 SS mesh was also studied. Using aqueous droplets of different pH values (pH = 0, 1, 4, 7, 9, 12, and 14), the WCA measurements did not change and were higher than 150°. This showed that the superhydrophobic mesh was resistant to corrosive media, with pH having no effect on the wetting properties of the surface due to its functional group (the carboxylate) not being ionizable as it is bonded to the iron oxide surface. Furthermore, the laurate molecules that were aliphatic chains were branched, which meant that they were in contact with water and showed better hydrophobicity.^57^ The superhydrophobic mesh was also placed in contact with hexane for 10 min, which did not affect its superhydrophobic properties. Additionally, to study the sustainability of the mesh and to confirm that the mesh did not dissolve, it was placed in contact with the aqueous phase for 24 h. ICP-OES analysis of the solution showed that the constituent elements of the 304 SS mesh and the iron oxide layer were not affected by the presence of water, with no iron being detected in the solution. This indicated a high degree of sustainability of the superhydrophobic 304 SS mesh as well as the presence of a superhydrophobic coating that prevented the oxidation of the mesh due to the air layer between the coating and the water. It is very important to take into account that the methods for MP removal usually present fouling of the pollutants such as the MP themselves, which directly limits their applications. Herein, as the surface was also self-cleaning, the pollutants could be easily removed, improving the ability to clean the mesh without the need for further treatments. Moreover, the UWOCA of the 304 SS mesh was calculated, which revealed underwater superoleophilicity. The UWOCA can be determined with the Bartell–Osterhof equation (eq 1), as follows:
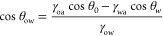
1where θ_ow_, θ_w_, and θ_o_ are the UWOCA, static WCA, and static OCA, respectively, while γ_oa_, γ_wa_, and γ_ow_ represent the surface free energy of oil–air, water–air, and oil–water, respectively. As hexane was used as the oil phase, the values used to determine the different surface free energy were those of the hexane–water interfacial tension (γ_ow_ = 50.25 mN/m), hexane–air (γ_oa_ = 18.43 mN/m), and water–air interface (γ_wa_ = 72.0 mN/m). For a surface to behave as superoleophilic in underwater conditions, the UWOCA must be <10°. The calculated value of θ_ow_ for hexane was 3 ± 2°, demonstrating that the modified 304 SS mesh was highly superoleophilic underwater too. This was consistent with the measured UWOCA revealing that the 304 SS mesh presented superoleophilicity underwater and that the organic phase remained adhered to the surface because the hierarchical structure produced by the chemical etching was completely filled with hexane, with the air phase removed due to the higher affinity of the substrate for hexane than for water.

**Figure 4:**
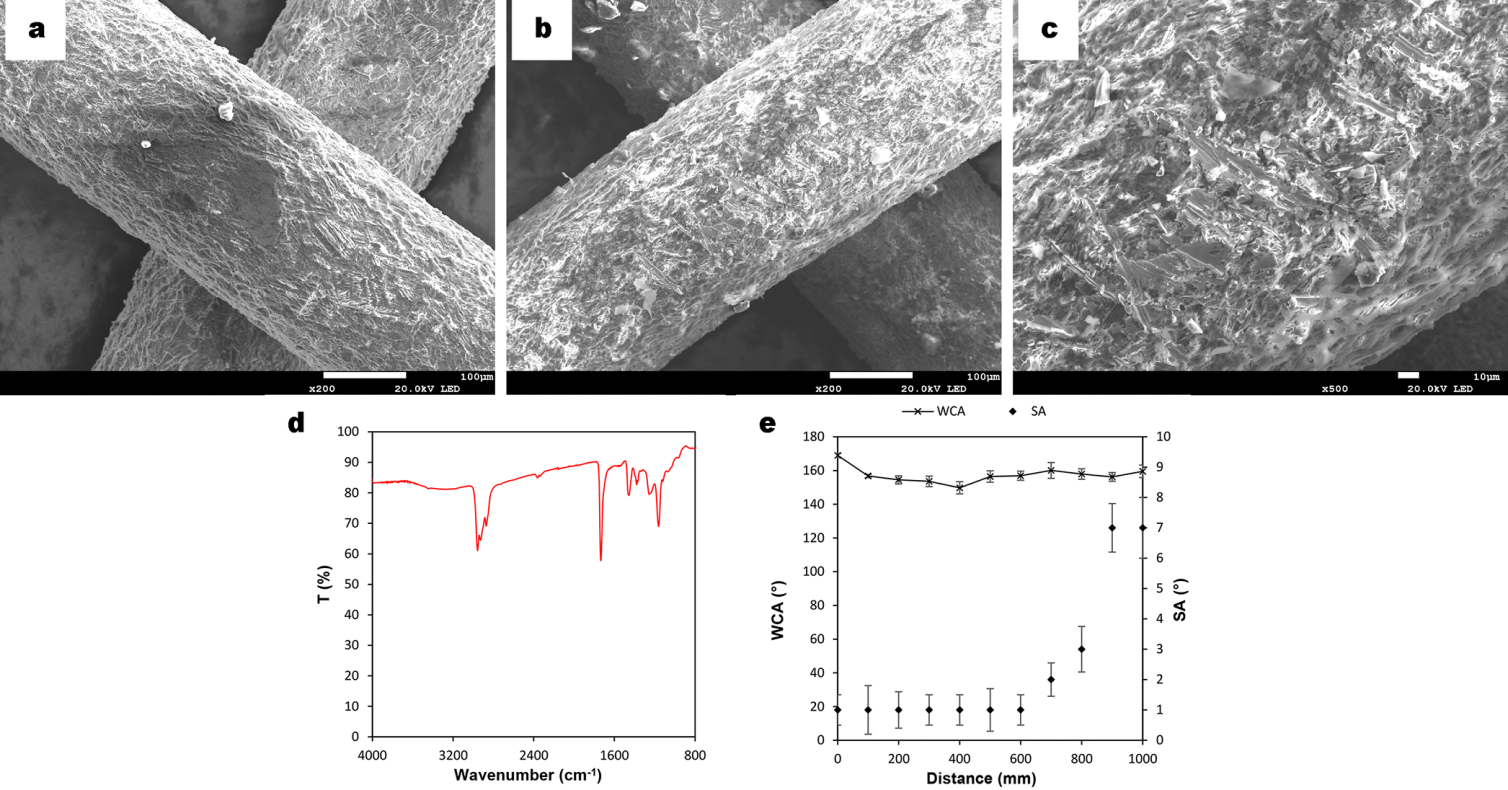
Durability test carried out with the SiC P1200 abrasive paper for 10 cycles at 5 kPa: (a) a micrograph of the superhydrophobic 304 SS mesh where the uppermost area was slightly removed, (b) the uppermost area presenting some scratches, (c) its magnification, (d) ATR-FTIR spectroscopy at the surface level showing the presence of the carboxylate functional group, and (e) changes in the WCA and SA during the durability test.

The robustness of the modified 304 SS mesh was also determined. As shown in Figure 4a–c, the upper areas of the mesh that were in contact with the abrasive paper appeared damaged after applying 5 kPa of pressure throughout the whole surface. In fact, there were two types of damages. On the one hand, the uppermost area of the superhydrophobic mesh was slightly removed and the hierarchical structure was no longer present (Figure 4a). On the other hand, the hierarchical structure could be observed in the uppermost areas, but there were some scratches (Figure 4b), magnified in Figure 4c, which were caused by the SiC abrasive paper. Even though these areas were affected, the wettable properties were not severely affected and the modified 304 SS mesh still presented superhydrophobic properties. Additionally, the surface was analyzed by ATR-FTIR spectroscopy to confirm the presence of the carboxylate group at the surface level (Figure 4d). The spectrum revealed the presence of the characteristic bands: there were sharp bands between ca. 3000 cm^–1^ and ca. 2800 cm^–1^ corresponding to v_as_CH_3_, v_as_CH_2_, and v_s_CH_2_–CH_2_, respectively, from the alkyl chain and a more intense band at ca. 1700 cm^–1^ corresponding to vC=O. Moreover, semiquantitative EDS (wt %) showed 41.8% Fe (Kα = 6.4 eV), 15.3% C (Kα = 0.277 eV), 12.7% O (Kα = 0.5 eV), 10.9% Mn (Kα = 5.9 eV), 8.3% Cr (Kα = 5.4 eV), 6.9% Cu (Kα = 8.1 eV), 1.7% Si (Kα = 1.739 eV), and 1.5% Ni (Kα = 7.5 eV) as well as minor elements (Figure S2). These results confirmed the presence of iron oxides (γ-Fe_2_O_3_ and α-FeOOH) after the abrasive test. The reason for this behavior was associated with the mesh surface that, despite the damage caused by the abrasive paper, still presented the hierarchical structure obtained after chemical etching and the liquid-phase deposition of lauric acid. Moreover, the carboxylate group that led to the formation of iron laurate retained a low surface free energy and water droplets did not adhere to the etched surface.

These results revealed that, under abrasive conditions, the surface remained highly superhydrophobic as the WCA almost did not change during the abrasive test. Moreover, after 600 mm of the abrasive test, the SA increased up to 7 ± 1° and then remained constant (Figure 4e). Hence, there were no pinning sites introduced on to the hierarchical structure, indicating that the superhydrophobic 304 SS mesh was still highly homogeneous in its chemical composition and also strongly robust after the test.^58−60^

### Microplastic Removal

**Figure 5:**
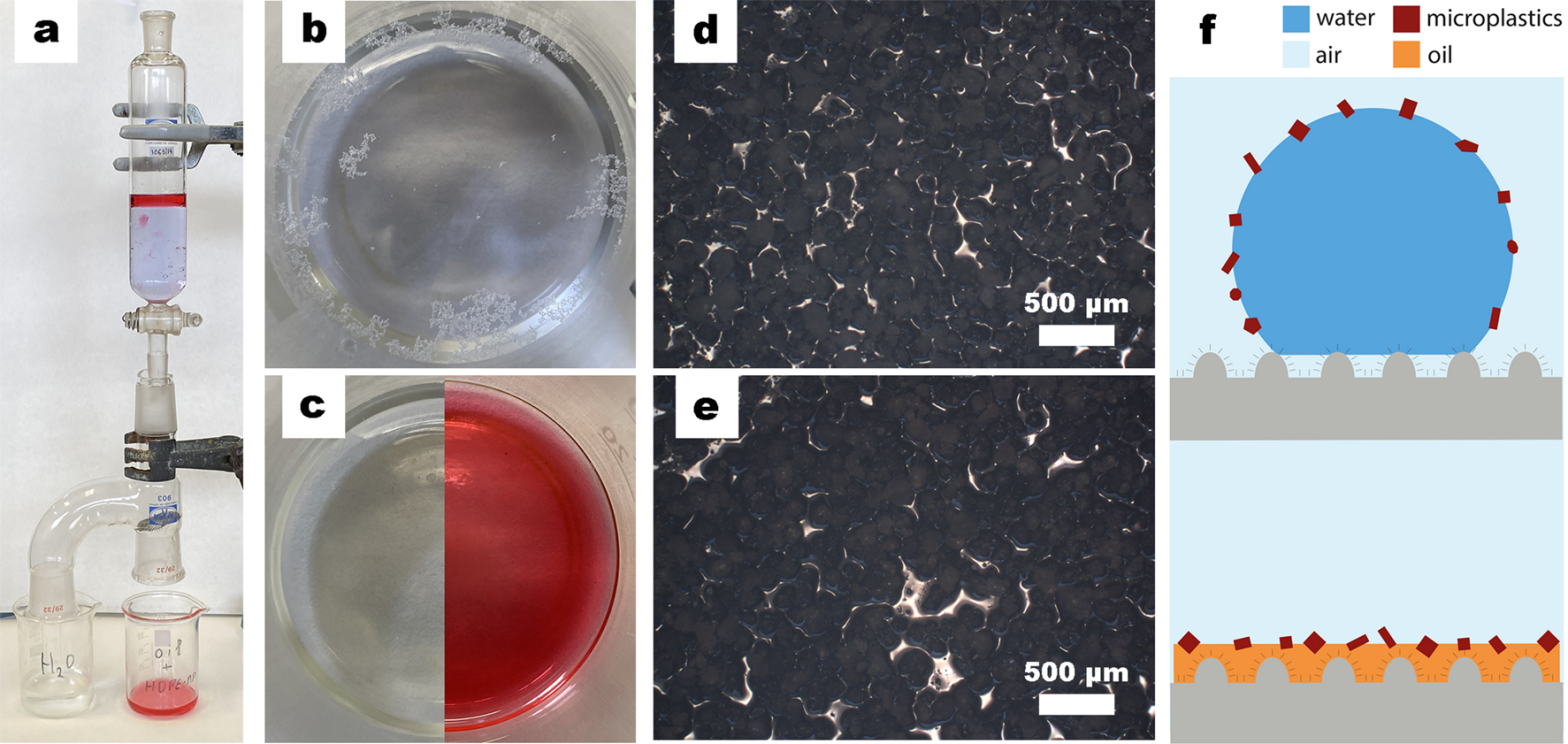
MP separation process: (a) laboratory-made device containing water (blue) and hexane (red) used for the removal of HDPE-MP; (b) the aqueous phase with HDPE-MP; (c) the aqueous phase (left) without the presence of HDPE-MP and the organic phase (right) containing HDPE-MP and hexane; HDPE-MP (d) before the separation process and (e) after the process, with the size of the MP not affected by the organic solvent, and (f) schematic showing the migration of MP from the water–air interface to the oil-air phase because of the superwettability properties.

The HDPE-MP removal process was carried out in a laboratory-made device prepared for this purpose (Figure 5a). This device was designed to simulate a possible system for the real removal of MP based on oil/water separation methods involving superhydrophobic materials.^61−64^ Initially, HDPE-MP were found randomly distributed in the aqueous phase, easily observable at the water–air interface (Figure 5b). The organic phase (hexane) was then added and both phases were stirred slightly. The mixture was then added into a dropping funnel, with a stopcock used to carefully control the amount of phase added through the inverted Claisen adapter containing the superhydrophobic mesh. Once the aqueous phase came in contact with the superhydrophobic mesh, the water droplets slid and were collected in the beaker on the left side, while the organic phase was collected through the mesh containing the HDPE-MP (Figure 5c, left to right). During this process, MP adhered to the surface of the 304 SS mesh and were collected in the corresponding beaker. The HDPE-MP size was not affected by the removal process (the sizes before and after each removal step were exactly the same) (Figure 5d, e). Moreover, HDPE-MP did not dissolve or swell in the presence of hexane. This is especially important because, in some industrial wastewater treatment methods operating under a water pressure (such as membranes), MP usually break down into smaller particles and can pass through the membrane and enter the environment again, limiting their application. In addition, the reusability of the superhydrophobic 304 SS mesh was evaluated by repeating the HDPE-MP removal process up to 15 times and measuring the WCA, SA, and OCA after each experiment. In all cases, the WCA remained slightly constant around 169°, the SA did not vary, and the OCA was 0° after each removal process. These results were in agreement with those from the durability test of the surface at different pH values and in the presence of hexane. This revealed that the mesh can be repeatedly used several times and shows high durability. To study how the removal process affected the superhydrophobic 304 SS mesh, the surface was characterized using FESEM-EDS, ATR-FTIR spectroscopy, and HR-XPS (Figure S3). As shown in the FESEM micrograph (Figure S3a), surface morphology was not affected by the oil or HDPE-MP and the cavities remained the same as before the removal of oil and HDPE-MP. Additionally, semiquantitative EDS (wt %) showed 45.3% Fe (Kα = 6.4 eV), 18.2% C (Kα = 0.277 eV), 12.0% O (Kα = 0.5 eV), 11.2% Mn (Kα = 5.9 eV), 8.3% Cr (Kα = 5.4 eV), 1.7% Si (Kα = 1.739 eV), 1.4% Ni (Kα = 7.5 eV), 1.3% Cu (Kα = 8.1 eV), and P as a minor element (Figure S3b). ATR-FTIR spectroscopy (Figure S3c) and HR-XPS (Figure S3d–f) showed that the chemical composition at the surface level was similar to that before the process. These results confirmed that after the HDPE-MP removal process, iron oxides (γ-Fe_2_O_3_ and α-FeOOH) were still present at the surface and iron laurate was the main surface chemical compound, explaining why the surface was still superhydrophobic.

As previously shown in the contact angle measurements, HDPE-MP presented more affinity for the hexane phase than for the aqueous phase. The MP showed an extremely low OCA and high WCA, which are characteristic of the intrinsic hydrophobicity of polymeric materials due to their low surface free energy. It is this difference in the wettability properties of MP between the two phases (oil and water), as well as the extreme repellency of the superhydrophobic mesh to water and its superoleophilic properties in air and under water, that leads to the separation and subsequent removal of MP. The migration of MP from water to the oil phase drives the separation process and allows the 304 SS mesh to capture HDPE-MP (Figure 5f).

To further evaluate the ability to remove MP, the removal efficiency (η) was determined using eq 2:
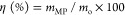
2where *m*_MP_ is the removed mass of HDPE-MP and *m*_o_ is the mass of HDPE-MP before the removal process corresponding to the mass added to the aqueous phase. The process completely removed HDPE-MP, with η = 99% for all the amounts of MP used. It also simultaneously removed 100% of the organic phase for a concentration of 2 mg/mL HDPE-MP in hexane. The efficiency to remove MP was also studied up to 15 times to determine if the process caused a decrease in the ability to remove HDPE-MP. The results demonstrated that the efficiency was not affected. In all cases, 99 ± 0.5% of the HDPE-MP and 100% of the organic phase were removed. This confirmed that the wetting properties of the mesh were the main factor influencing the removal efficiency. While water droplets could easily slide, the organic phase containing the HDPE-MP was spread throughout the whole mesh. Additionally, the relationship between the HDPE-MP size and the size of the mesh pore (0.5 mm^2^) should be considered. The 304 SS mesh presents superhydrophobic properties, indicating that a water droplet can easily slide through the whole mesh, even over the pores of the mesh, without getting trapped. Meanwhile, the organic phase completely permeates the surface containing the HDPE-MP. Here, MP can behave in two different ways: first, they remain adhered to the mesh and then to the different filaments (diameter = 270 μm) due to the superoleophilic properties of the surface; and second, once the mesh is saturated with oil, HDPE-MP pass through the mesh pores along with the oil due to gravity.^65−67^ This behavior could explain why the removal of HDPE-MP with different sizes was not affected by the mesh pore size. At the beginning, HDPE-MP adhere to the mesh filaments and can pass through the mesh pores. It is noteworthy to mention that the size of the MP is smaller than the mesh pore size. Therefore, they can easily pass through the pores without stopping the oil flux, keeping the separation constant. The removal process was also carried out with polypropylene MP, which produced results remarkably similar to those of HDPE-MP, with removal efficiencies close to 99%. This demonstrated that the use of an organic phase with the superhydrophobic surface improves the removal of MP.

Among the available methods used to completely remove HDPE-MP from water, meshes or membranes present elimination efficiencies close to 99%. Ultrafiltration and Al/Fe-based coagulation can also remove polyethylene MP from wastewater, revealing that filtration can completely remove MP.^68^ Membrane bioreactors involving rapid sand filtration, dissolved air flotation and biological catalysts have also been reported to remove 99% of MP.^69^ Furthermore, a method using H_2_O_2_ as a digestion agent as well as the addition of acetic acid and an adsorption separation technique was demonstrated to recover MP at a rate of 98.0%.^70^ Finally, disc filtration has been used to remove polyethylene MP, showing a 90% elimination of those MP.^71^ Despite the extremely high removal efficiencies, these methods usually present fouling, which is clearly a disadvantage that is avoided by using the superhydrophobic 304 SS mesh. Moreover, the use of superhydrophobic materials allows separation in only a single step.

Once we determined that the superhydrophobic surface could remove HDPE-MP by taking advantage of their wetting properties, particularly superoleophilicity, it was important to discuss the process itself from a surface point of view. In fact, the physical properties of MP as well as their surface chemistry can be similar to those shown by colloids.^72,73^ Before the separation, both phases were mixed and hexane was located at the top of the mixture due to its lower density (ρ_o_ = 0.655 g/mL) compared to that of water (ρ_w_ = 0.997 g/mL). The mixture was then stirred to promote the transport of HDPE-MP from the aqueous phase to the organic one. This was a gravity-driven transport method that can happen when ρ_o_ < ρ_w_ and, at the same time, ρ_p_ > ρ_o_, where ρ_p_ is the HDPE-MP particle density (ρ_p_ = 0.951 g/L). Additionally, HDPE-MP remained at the oil/water interface without penetrating any fluid.

It is well-known that polymers are hydrophobic (WCA > 90°), which explains their natural tendency to separate from water, thereby increasing the difficulty of removing them directly from aqueous systems. Consequently, the two main characteristics related to the surface phenomena in MP should be taken into account: (i) the binding energy (Δ*E*) of the MP to the liquid–vapor or solid–liquid interfaces and (ii) interparticle interactions.

On the one hand, the wettability of MP depends on their chemical composition, their density, as well as the presence of functional groups on the surface. Thus, assuming an aqueous media and a spherical shape of the MP, the Δ*E* of MP is defined as follows (eq 3):^74−77^
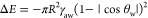
3where γ_aw_ is the surface free energy of the air–water phase, θ_w_ is the static WCA on the MP surface, and *R* is the radius of the MP. As *R* decreases, Δ*E* decreases nonlinearly. This means that MP increase their hydrophilic character and are more difficult to remove from water because of their low binding energy. On the other hand, in aqueous solution, MP present different interparticle interactions that depend on the MP size, the chemical composition of MP and the medium in which the MP are found.^78,79^ These interactions are defined by the DLVO theory. The net interaction can be calculated as follows (eq 4):
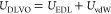
4where *U*_EDL_ is the electrostatic interaction and *U*_vdW_ is the van der Waals forces. When MP are found in water, their surface presents a distribution of charges caused by the dissociation of surface groups such as hydroxyls (−OH → −O^–^) and carboxylates (−COOH → −COO^–^). This phenomenon is balanced by oppositely charged ions or an induced dipole in the case of water molecules generating a diffuse charge region around the MP particles. The combination of both, which are the charged surface of MP and the counterion region, leads to the formation of an electric double layer that causes osmotic repulsion due to the overlap of both charge regions. As a result, this electric double layer prevents MP aggregation and increases stability at longer distances between the MP particles. In fact, these interactions increase the *U*_EDL_, while *U*_vdW_ are important at short interparticle distances but negligible at long interparticle distances.

Considering the surface mechanisms described before (Δ*E* and the DLVO theory), the removal of HDPE-MP can be explained as follows. In the experiments carried out, HDPE-MP migrate from water to hexane because of their superoleophilic properties. As shown with eq 3, the lower the Δ*E*, the higher the dispersibility of the MP. By contrast, at higher values of Δ*E*, MP tend to aggregate, making it easier to remove them. In fact, as HDPE-MP are first found in water and then in hexane, eq 3 can be modified as follows:
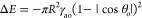
5where γ_ao_ is the surface free energy of the air–oil (hexane in this case) interface, θ_o_ the OCA on the MP surface and R the radius of the MP (as in the previous equation). Therefore, Δ*E* can be calculated for both phases. For water, γ_aw_ = 72.0 mN/m and WCA = 136°, while for hexane, γ_ao_ = 18.3 mN/m and OCA = 0°. In the experiment carried out here, the R of the HDPE-MP was constant at 133 μm, while the WCA, OCA and the surface free energy were the variables in the equation. The result of the equation for the aqueous phase was −1.917 × 10^10^*k*_b_T. For the hexane phase, as the OCA was 0°, the equation was null, therefore Δ*E* was ∼0 *k*_b_T. These binding energy values demonstrate that HDPE-MP in water present low binding energy and high dispersibility as predicted. When the HDPE-MP are found in the organic phase, the binding energy is 0 and the MP tend to aggregate more than in the aqueous phase. It is necessary to better explain the reason why the solid pollutants tend to aggregate in oil rather than in water. As the media changes from an aqueous phase to an organic one, the electrostatic interactions between the MP are negligible as water molecules are no longer present and cannot ionize the surface functional groups of the MP. Thus, the electric double layer is not generated. In this scenario, the interparticle interactions between the MP change because the Coulombic interactions between the surface and the charge region are no longer present. When HDPE-MP are in the organic phase, van der Waals forces are prominent because the chemical composition of HDPE-MP favors their aggregation. Once the HDPE-MP aggregate, the organic phase containing the HDPE-MP can be easily removed with the 304 SS mesh through its superoleophilic properties.

## Conclusions

Herein, we show that, after modifying the surface of a 304 SS mesh through oxidizing conditions (FeCl_3_/HCl/H_2_O_2_) followed by the liquid-phase deposition of lauric acid, the surface free energy of the whole system changed, showing superhydrophobicity (169°) and superoleophilicity (0°). Additionally, the wetting properties of HDPE-MP were measured, which showed hydrophobicity (136°) and superoleophilicity (0°). Taking advantage of these properties, the ability of the superhydrophobic mesh to remove HDPE-MP was evaluated. In a laboratory-made system, HDPE-MP migrated from the aqueous phase to the organic phase, with the MP removed via the superoleophilic properties of the modified 304 SS mesh. Furthermore, the removal behavior was explained by the binding energy of the HDPE-MP (Δ*E*) as well as by DLVO theory. Both theories propose that, due to the higher affinity of HDPE-MP for the oil phase (hexane), the MP aggregate better in oil than in the aqueous phase where MP tend to disperse. Due to the repulsive cloud formed throughout the surface of the MP in water, dispersion is enhanced, while in the organic phase MP tend to aggregate through van der Waals interactions. These results demonstrate that superhydrophobic materials can separate MP from water, leading to an innovative application of these systems to tackle a global issue. In addition, by elucidating the theoretical basis of the removal process based on the colloidal properties of MP, further research should be carried out to expand these applications using different materials, geometries, and complex organic solvents.

## Supporting Information Available

Semiquantitative EDS after chemical etching and LPD of lauric acid; semiquantitative EDS after abrasive paper test; characterization of the superhydrophobic 304 SS mesh after the removal of pollutants: (a) FESEM surface morphology, (b) semiquantitative EDS, and (c) ATR-FTIR (PDF)
