# Supplementary material for: Superhydrophobic 304 Stainless Steel Mesh for the Removal of High-Density Polyethylene Microplastics
Source: Langmuir. 2022 Apr 25;38(18):5943–53. doi: 10.1021/acs.langmuir.2c00803 (PMC9097532; doi:10.1021/acs.langmuir.2c00803)
Supplement: Supplementary file 1 — la2c00803_si_001.pdf [file la2c00803_si_001.pdf]

Superhydrophobic 304 stainless steel mesh for the removal of high-density polyethylene microplastics

Oriol Rius-Ayra*, Alisiya Biserova-Tahchieva, Victor Sansa-López, and Núria Llorca-Isern

CPCM Departament de Ciència dels Materials i Química Física, Facultat de Química, Universitat de Barcelona, Martí i Franquès 1 - 11, 08028 Barcelona, Spain

*Corresponding author: oriolriusayra@ub.edu

Table of Contents

Figure S1. Semiquantitative EDS after chemical etching and LPD of lauric acid. S2

Figure S2. Semiquantitative EDS after abrasive paper test. S2

Figure S3. Characterization of the superhydrophobic 304 SS mesh after the removal of pollutants: a) the surface morphology, b) Semiquantitative EDS, c) ATR-FTIR and d) HR-XPS. S3

References. S6

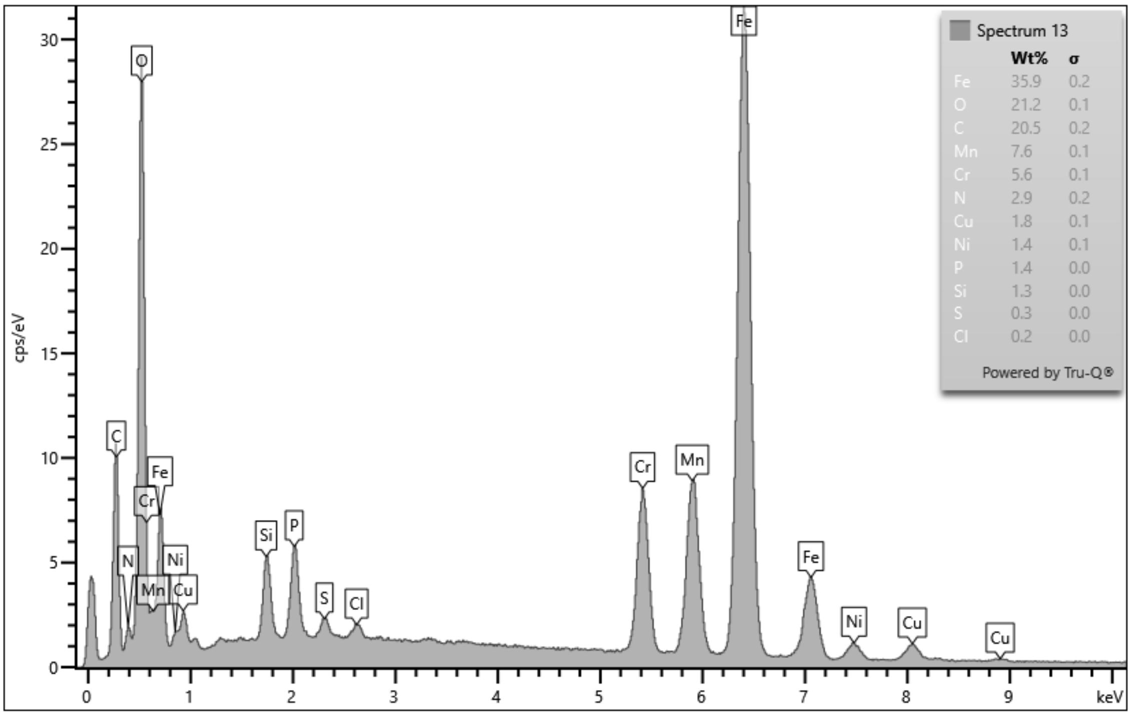

Figure S1. Semiquantitative EDS after chemical etching and LPD of lauric acid.

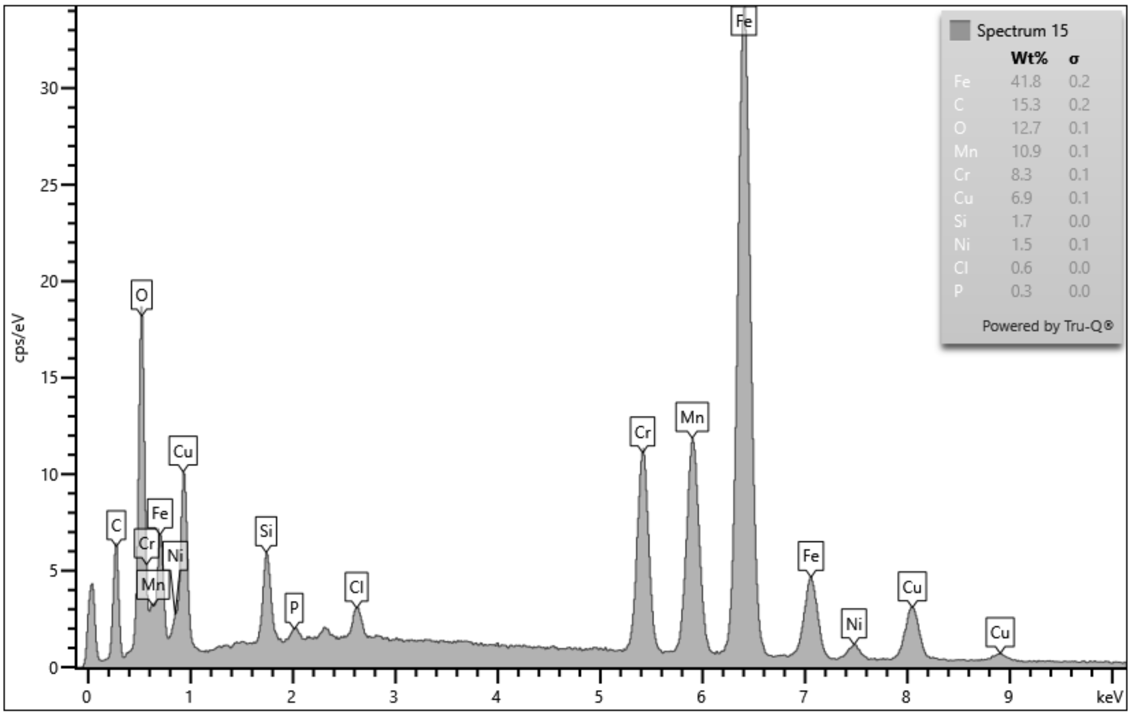

Figure S2. Semiquantitative EDS after abrasive paper test.

a)

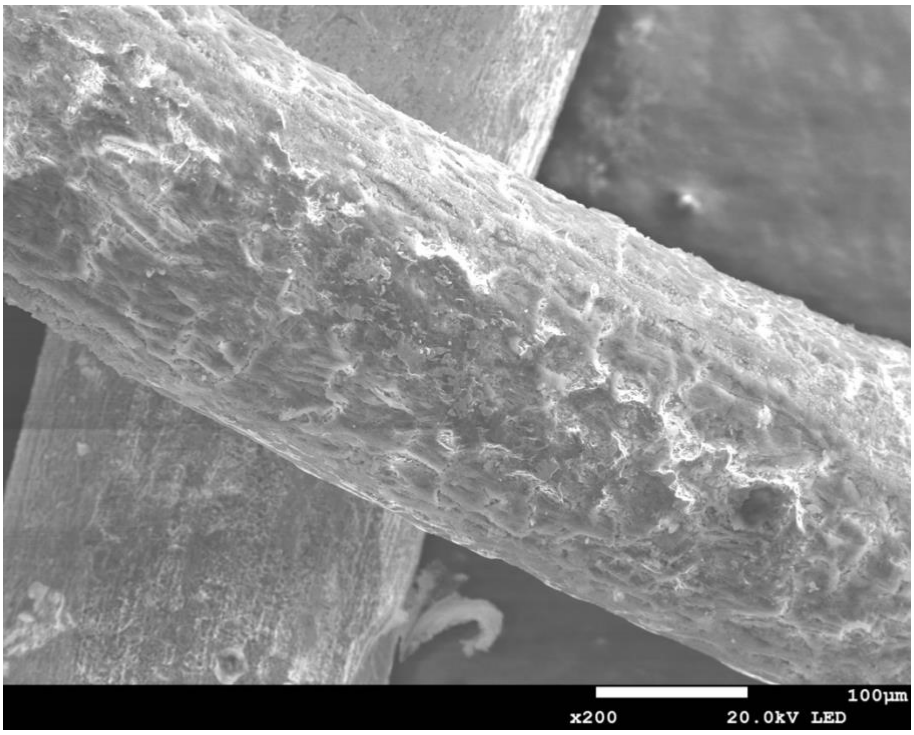
100µm
x200
20.0kV LED

b)

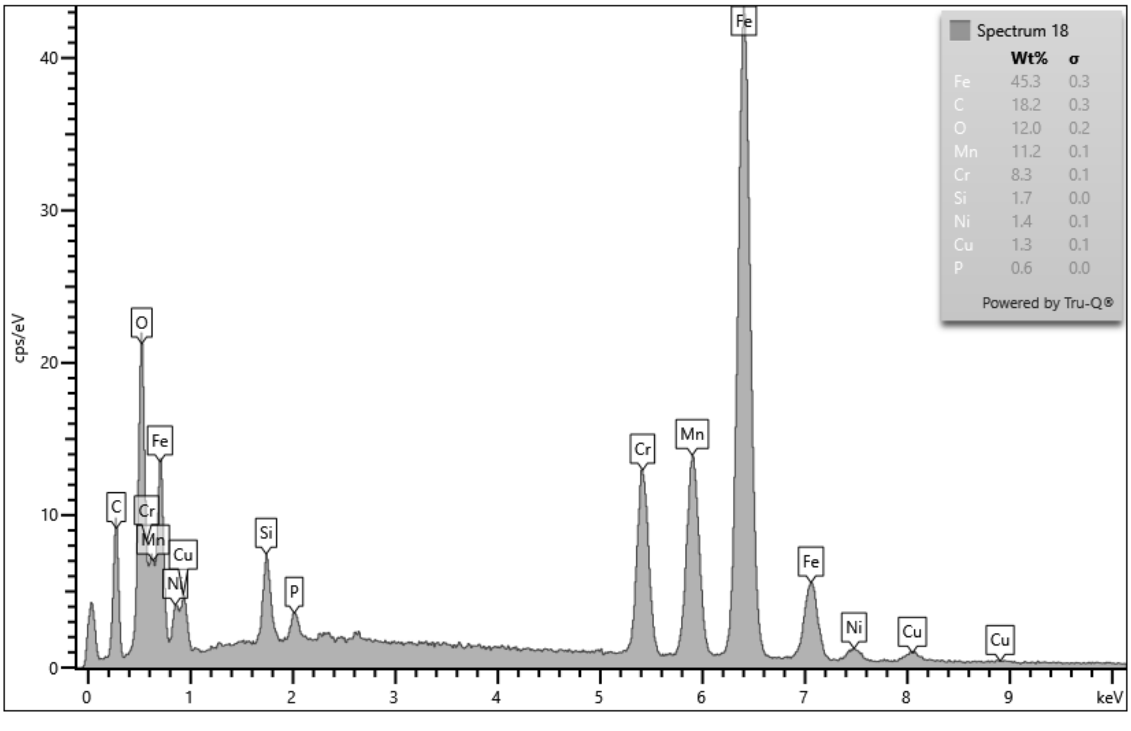
Spectrum 18
Wt% σ
Fe 45.3 0.3
C 18.2 0.3
O 12.0 0.2
Mn 11.2 0.1
Cr 8.3 0.1
Si 1.7 0.0
Ni 1.4 0.1
Cu 1.3 0.1
P 0.6 0.0
Powered by Tru-Q®
cps/eV
keV
Fe
O
Fe
C
Cr
Mn
Cu
Ni
Si
P
Cr
Mn
Fe
Ni
Cu
Cu

c)

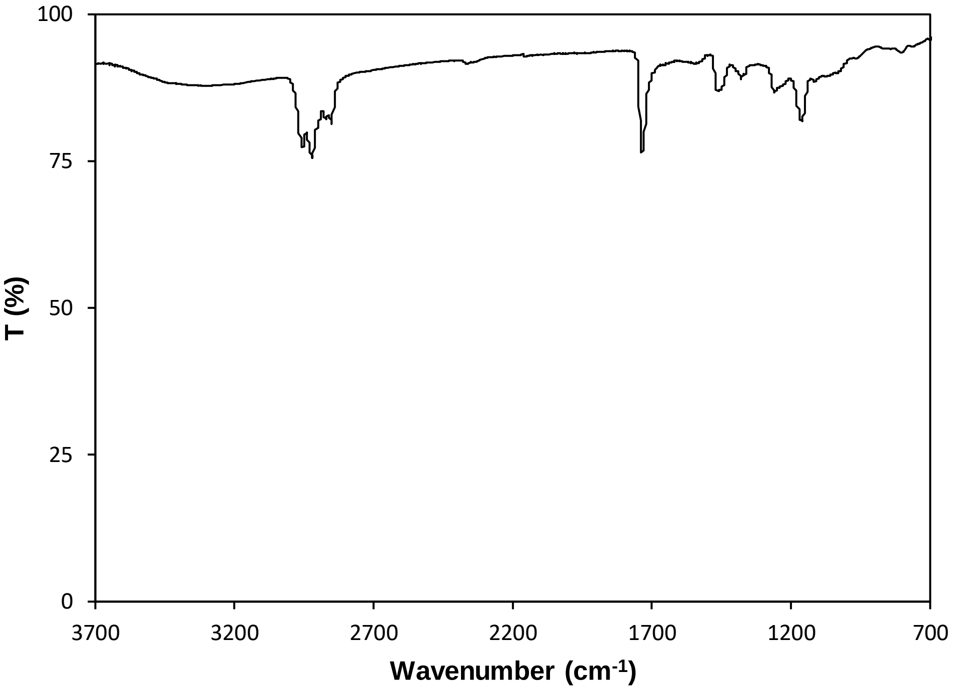
100
75
50
25
0
T (%)
3700
3200
2700
2200
1700
1200
700
Wavenumber (cm-1)

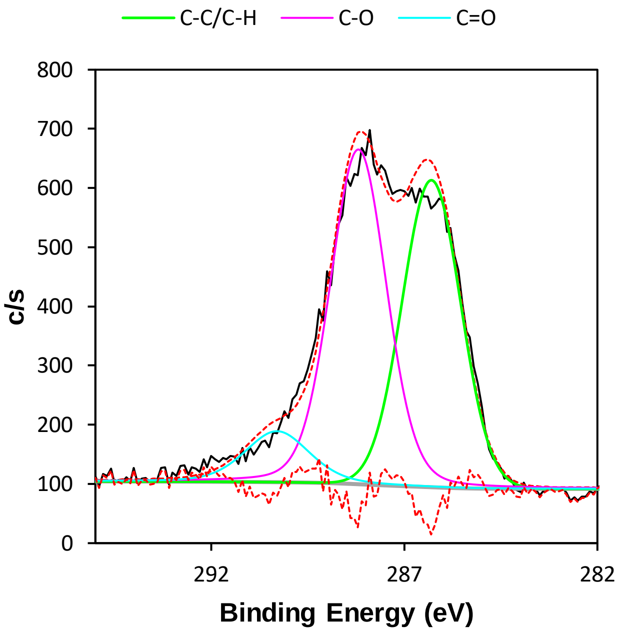
C-C/C-H
C-O
C=O
d)
800
700
600
500
400
300
200
100
0
c/s
292
287
282
Binding Energy (eV)

e)

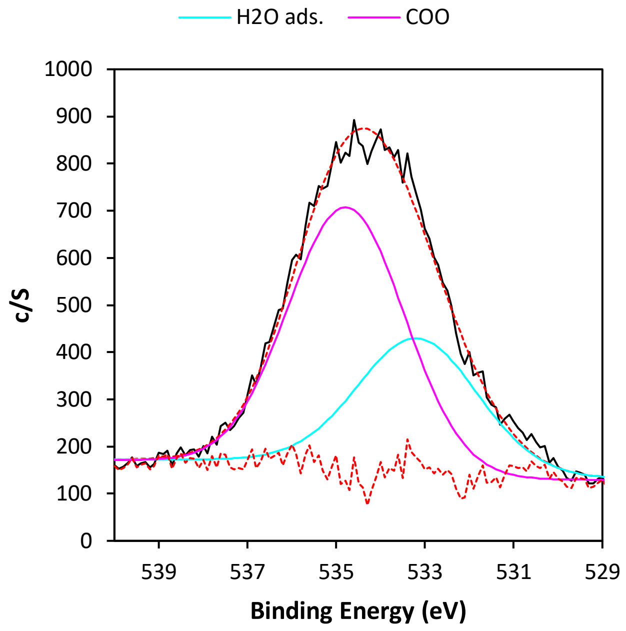

f)

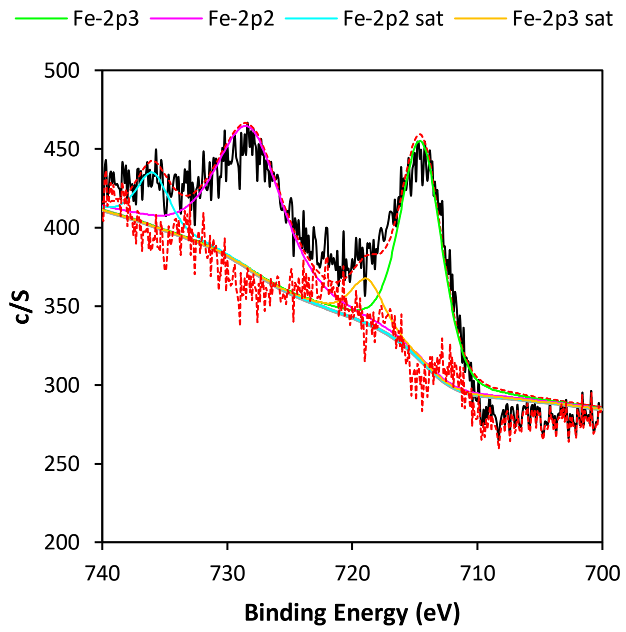

Figure S3. Characterization of the superhydrophobic 304 SS mesh after the removal of pollutants: a) the surface morphology remained similar compared to the surface before the removal process, b) Semiquantitative EDS, c) ATR-FTIR were between ca. 3000 cm^{-1} and ca. 2800 cm^{-1} there were the corresponding bands to $v_{as}CH_3$, $v_{as}CH_2$ and v_sCH_2-CH_2 respectively from the alkyl chain and a band ca. 1700 cm^{-1} corresponding to vC=O, *d) HR-XPS of C-1s, e) HR-XPS of O-1s and f) HR-XPS of Fe-2p*

The ATR-FTIR showed the characteristic bands of the carboxylate with sp^3 carbon and carbonyl (C=O) signals (Figure S3 c) [1–3]. In case of HR-XPS, the C-1s showed three deconvolutions (Figure S3 d) at 286 eV (C-C/C-H), 288 eV (C-O) and at 290 eV (C=O) [4]. The O-1s there were two deconvolutions (Figure S3 e) at 533 eV corresponding to the adsorbed water and at 534 eV assigned to the carboxylate functional group (COO^-) [5–8]. Finally, Fe-2p showed four deconvolutions (Figure S3 f) at 715 eV (γ-Fe_2O_3), at 728 eV (α-FeOOH) and two more peaks corresponding to the satellites at 719 eV (Fe-2p3/2) and at 735 eV (Fe-2p1/2) [5,7,8].

References

(1) Gadermann, M.; Preston, T.; Troster, C.; Signorell, R. Characterization of Palmitic and Lauric Acid Aerosols from Rapid Expansion of Supercritical CO2 Solutions. *Mol. Phys.* **2008**, *106* (7), 945–953. https://doi.org/10.1080/00268970802020355.

(2) Uma Maheswari, J.; Krishnan, C.; Kalyanaraman, S.; Selvarajan, P. Growth and Characterization of an Organic Nonlinear Optical Material-Lauric Acid Crystal. *Mater. Res. Express* **2016**, *3* (10), 2–3. https://doi.org/10.1088/2053-1591/3/10/105101.

(3) Jiesheng, L.; Yuanyuan, Y.; Xiang, H. Research on the Preparation and Properties of Lauric Acid/Expanded Perlite Phase Change Materials. *Energy Build.* **2016**, *110*, 108–111. https://doi.org/10.1016/j.enbuild.2015.10.043.

(4) Men, S.; Jiang, X.; Xiang, X.; Sun, G.; Yan, Y.; Lyu, Z.; Jin, Y. Synthesis of Cellulose Long-Chain Esters in 1-Butyl-3-Methylimidazolium Acetate: Structure-Property Relations. *Polym. Sci. - Ser. B* **2018**, *60* (3), 349–353. https://doi.org/10.1134/S1560090418030144.

(5) Biesinger, M. C.; Payne, B. P.; Grosvenor, A. P.; Lau, L. W. M.; Gerson, A. R.; Smart, R. S. C. Resolving Surface Chemical States in XPS Analysis of First Row Transition Metals, Oxides and Hydroxides: Cr, Mn, Fe, Co and Ni. *Appl. Surf. Sci.* **2011**, *257* (7), 2717–2730. https://doi.org/10.1016/j.apsusc.2010.10.051.

(6) Trinh, Q. T.; Bhola, K.; Amaniampong, P. N.; Jérôme, F.; Mushrif, S. H. Synergistic Application of XPS and DFT to Investigate Metal Oxide Surface Catalysis. *J. Phys. Chem. C* **2018**, *122* (39), 22397–22406. https://doi.org/10.1021/acs.jpcc.8b05499.

(7) He, Y.; Yao, X.; Dong, Q.; Batista, V. S.; Brudvig, G. W.; Yang, K. R.; Li, W.; Wang, D. Facet-Dependent Kinetics and Energetics of Hematite for Solar Water Oxidation Reactions. *ACS Appl. Mater. Interfaces* **2018**, *11*, 5616–5622.

https://doi.org/10.1021/acsami.8b05190.
(8) Kim, Y. Y.; Min, K.; Piraman, S.; Sundar, S.; Mariappan, R. Nanospheres and Nanoleaves of γ-Fe_2O_3 Architecturing for Magnetic and Biomolecule Sensing Applications. *Sensors Actuators B Chem.* **2016**, *234*, 386–394. https://doi.org/10.1016/j.snb.2016.04.168.
